# Burden and trend of colorectal cancer in 54 countries of Africa 2010–2019: a systematic examination for Global Burden of Disease

**DOI:** 10.1186/s12876-022-02275-0

**Published:** 2022-04-25

**Authors:** Atalel Fentahun Awedew, Zelalem Asefa, Woldemariam Beka Belay

**Affiliations:** 1 Department of Surgery, SoM, Addis Ababa University, P.Box:1176, Addis Ababa, Ethiopia; 2 Department of Surgery, Debre Tabor Hospital, Debre Tabor, Ethiopia

**Keywords:** Colorectal, Cancer, Africa, Burden

## Abstract

**Background:**

Colorectal cancer plays significant role in morbidity, mortality and economic cost in Africa.

**Objective:**

To investigate the burden and trends of incidence, mortality, and disability-adjusted life-years (DALYs) of colorectal cancer in Africa from 2010 to 2019.

**Methods:**

This study was conducted according to Global Burden of Disease (GBD) 2019 analytic and modeling strategies. The recent GBD 2019 study provided the most updated and compressive epidemiological evidence of cancer incidence, mortality, years lived with disability (YLDs), years of life lost (YLLs), and DALYs.

**Results:**

In 2019, there were 58,000 (95% UI: 52,000–65,000), 49,000 (95% UI: 43,000–54,000), and 1.3 million (95% UI: 1.14–1.46) incident cases, deaths and DALYs counts of colorectal cancer respectively in Africa. Between 2010 and 2019, incidence cases, death, and DALY counts of CRC were significantly increased by 48% (95% UI: 34–62%), 41% (95% UI: 28–55%), and 41% (95% UI: 27–56%) respectively. Change of age-standardised rates of incidence, death and DALYs were increased by 11% (95% UI: 1–21%), 6% (95% UI: − 3 to 16%), and 6% (95% UI: − 5 to 16%) respectively from 2010 to 2019. There were marked variations of burden of colorectal cancer at national level from 2010 to 2019 in Africa.

**Conclusion:**

Increased age-standardised death rate and DALYs of colorectal cancer indicates low progress in CRC standard care-diagnosis and treatment, primary prevention of modifiable risk factors and implementation of secondary prevention modality. This serious effect would be due to poor cancer infrastructure and policy, low workforce capacity, cancer center for diagnosis and treatment, low finical security and low of universal health coverage in Africa.

## Background

Colorectal cancer plays significant role in morbidity, mortality and economic cost. In 2019, Global Burden Disease study reported that CRC accounted for 1.8 million incidence cases, 0.9 million deaths, and 19 million DALYs worldwide [1]. According to GLOBACAN reported in 2020, colorectal cancer was responsible for more than 1.9 million new incident cases and 0.94 million deaths, making third and second rank for overall cancer incidence and mortality globally [2]. Global incidence cases of CRC doubled or more than doubled in 157 of 204 countries, and mortality due to CRC doubled of more doubled in 129 of 204 countries, pronounced increases were observed in low and Middle SDI countries from 1990 to 2019 [3]. Due to the rapid rising of global population size, aging and human economic development, burden of CRC is predicted to be 2.2 million new cases and 1.1 million cancer deaths by 2030 [4] and 3.2 million new incidence cases in 2040 [5]. This trend alarms all concern bodies to stand for prevention and control of CRC. CRC is the indicator of socioeconomic transition, epidemiological and demographic change. The current global evidence ascertains that trend of CRC has three patterns-rapidly rising in many LMICs which is associated with socioeconomic transition, stabilizing or decreasing in middle high and high income countries [1, 2, 4]. Development of CRC has associated with males and older age. Lifetime risk of CRC estimated approximately 4.4% of men (1 in 23) and 4.1% of women (1 in 25) [6]. Approximately 70% of CRC cases occur sporadic, whereas the remaining 12–35% and 5–7% are linked with familiar and genetic respectively [7, 8]. More than half (55%) of all CRCs have attributed to lifestyle factors, including an unhealthy diet, insufficient physical activity, high alcohol consumption, and smoking [6]. Global efforts have tried to alleviate serious effect of cancer, specifically major cancers such as CRC, breast, and cervical cancer. In 2012, World Health Assembly members agreed to reduce premature death from noncommunicable diseases (NCDs) by 25% by 2025 [9]. In 2015, United Nations (UN) Sustainable Development Goals planned to reduce NCD related premature mortality by one-third by 2030 [10]. Understanding the trend and variation in incidence, DALYs and mortality of colorectal cancer helps for public health experts, Professional experts, national policy makers and cancer prevention advocacy groups to bring evidence based decision in their countries and to evaluate the effectives, accessibility, affordability, and efficiency of interventions. GLOBACAN and GBD are the two studies that provide national, regional and global burden of cancers. Despite this evidence, burden of CRC in Africa and nations are not well narrated due to their compressive report. Therefore, considering the aforementioned issues, the present study provides regional and national incidence, mortality and DALYs for colorectal cancer in terms of counts, age-standardised rates, and percentage change for 54 countries from 2010 to 2019.

## Methods

The data used for analysis of this study was obtained from GBD2019 data tools (http://ghdx.healthdata.org/gbd-results-tool). The study conducted based on GBD2019 methodology framework and tools. The GBD study provides a standardised approach for estimating incidence, prevalence, and DALYs by cause, age, sex, year, and location for global, regions and countries. The incidence, DALYs and mortality for CRC reported as part of the Global Burden of Disease, injury, and risk factors study 2019. The GBD 2019 estimates provided evidences for 363 causes of non-fatal burden, 302 causes of deaths, and 87 risk factors in 204 countries and territories, 21 regions and 7 supper regions. The main sources of data used for GBD estimation were obtained from cancer registry, vital registration, sample registration system, and verbal autopsy [11]. There are three main standardised tools: Cause of Death Ensemble model (CODEm), spatiotemporal Gaussian process regression (ST-GPR), and DisMod-MI [11]. Cause of Death Ensemble model (CODEm) developed after stepwise data transformation of raw data. First, incidence and mortality data obtain from different sources are transformed into standardised format, categorize and registered. After standardised, cancer registry incidence data and cancer registry mortality data are mapped to GBD causes and standardized to the GBD age groups. Incidence and mortality data from cancer registries were processed before matching the same by cancer, age, sex, year, and location to generate crude mortality-to- incidence (MI) ratio. Finally, MI ratios estimates were estimated using a linear step mixed- effects model using the logit link function, in which healthcare access and quality (HAQ) index served as a covariate. The ST-GPR model has three main hyperparameters that control for smoothing across time, age, and geography. The final mortality estimates were produced using the Cause of Death Ensemble Model (CodeM) using crude mortality estimates as inputs along with other variables taken as covariates. DALYs of CRC was estimated using DisMod-MR 2.1 proportion model. The input of data for DisMod-MR 2.1 was procedure-related disability (ostomies) for all locations by age, sex, and year. Evidence from literature review narrated that an average of 58% of all ostomies are for colorectal cancer, so we multiplied the all-cause ostomies by 0·58 [12].

## Result

### Colorectal burden of Africa

In 2019, estimated incident new cases of colorectal cancer in Africa were 58,000 (95% UI: 52,000–65,000), with age-standardised 8.7 (95% UI: 8.–9.4) per 100,000 in both sexes. The incidence cases increased significantly from 40,000 (95% UI: 36,000–43,000) in 2010 to 58,000 (95% UI: 52,000–65,000) in 2019, which represented a percentage change of 48% (95% UI: 34–62%) and AAPC 4.4% (95% UI: 4.3–4.5%). Change of age –standardised incidence rate of CRC between 2010 and 2019 was 11% (95% UI: 1–21%) and AAPC was 1.1% (95% UI: 1–1.2%).

In 2019, estimated absolute number of deaths due to colorectal cancer in Africa was 49,000 (95% UI: 43,000–54,000), with age-standardised 8.1 (95% UI: 7.4–8.8) per 100,000. Between 2010 and 2019, deaths due to colorectal cancer increased from 35,000 (95% UI: 31,000–38,000) to 49,000 (95% UI: 43,000–54,000), which represented 41% (95% UI: 28–55%) and AAPC was 3.9% (95% UI: 3.8–4%). Change of age-standardised death rate of CRC between 2010 and 2019 was 6% (95% UI: − 3 to 16%) and AAPC was 0.7% (95% UI: 0.4–1%).

In 2019, estimated DALYs counts of colorectal cancer in Africa were 1.3 milion (95% UI: 1.14–1.46), with age-standardised 180 (95% UI: 160–200) per 100,000. The DALYs counts of colorectal increased significantly from 0.92 million (95% UI: 0.84–1.01) in 2010 to 1.3 million (95% UI: 1.1–1.6 in 2019, which represented 41% (95% UI: 27–56%) and AAPC was 3.7% (95% UI: 3.6–3.8%). Change of age-standardised DALYs rate of CRC between 2010 and 2019 was 6% (95% UI: − 5 to 16%) and AAPC was 0.6% (95% UI: 0.5–0.7%). For comparing purpose, we explored the trends of CRC in Europe, America, Asia and Global (Table 1).

**Table 1:** Comparing change of burden of colorectal cancer from 2010 to 2019

Regions	Incidence cases			Death counts			DALYs counts		
	Value (%)	95% UI (%)		Value (%)	95% UI (%)		DALYs (%)	95% UI (%)	
Global	32	24	41	27	20	33	23	16	30
Africa	48	34	62	41	28	55	41	27	56
America	28	15	41	26	22	31	24	20	29
Asia	46	32	62	37	25	49	31	19	43
Europe	13	2	24	10	5	14	5	1	10

Regions	ASIR			ASDR			Age standardised DALYs rate		
	Value (%)	95UI% (%)		Value (%)	95UI% (%)		Value (%)	95% UI (%)	
Global	2	− 4	9	− 3	− 8	2	− 3	− 8	3
Africa	11	1	21	6	− 3	16	6	− 5	16
America	0	− 9	11	− 1	− 5	2	− 1	− 5	3
Asia	9	− 1	20	0	− 8	9	1	− 8	10
Europe	− 1	− 10	9	− 6	− 10	− 2	− 7	− 11	− 2

### Distribution of Burden of CRC among sexes in Africa

In 2019, CRC accounted for 31, 00 (95% UI: 27,000–36,000), 2500 (95% UI: 22,000–29,000), and 6.9 million (95% UI: 6–7.9) incidence cases, deaths and DALYs counts among males in Africa respectively. In Africa, CRC accounted for 27, 00 (95% UI: 24,000–30,000), 2300 (21,000–26,000), and 6.1 million (95% UI: 5.2–7) incidence cases, deaths and DALYs counts respectively among females in 2019. In 2019, age-standardised rates of incidence cases, deaths, and DALYs of CRC were 10.6 (95% UI: 9.4–11.9), 9.2 (95% UI: 8.2–10.3), and 210 (95% UI: 180–230) per 100,000 in African males respectively, and 8.7 (95% UI: 7.7–9.7), 8 (95% UI: 7.1–9), and 170 (95% UI: 150–200) per 100,000 in African females respectively. In Africa, between 2010 and 2019, percentage change of incidence cases, death and DALYs counts of CRC were 48% (95% UI: 31–65%), 40% (95% UI: 23–56%), and 40% (95% UI: 23–57%) in males, respectively, and 47% (95% UI: 31–64%), 42% (95% UI: 27–58%), and 42% (95% UI: 26–60%) in females, respectively. From 2010 to 2019 in Africa, changes of age-standardised rate of incidence, death and DALYs were 12% (95% UI: 0–25%), 7% (95% UI: − 5 to 19%), and 6% (95% UI: − 6 to 18%) respectively in males, and 10% (95% UI: − 1 to 21%), 6% (95% UI: − 4 to 17%), and 6% (95% UI: − 6 to 18%) respectively in females. From 2010 to 2019 in Africa, the average annual percentage change (AAPC) of incidence cases was 4.4% (95% UI: 4.3–4.5) in males and 4.4% (95% UI: 4.3–4.5) in females while AAPC of age-standardised DALYs was 1.3% (95% UI: 1.1–1.4%) in males and 1% (95% UI: 0.9–1.1%) in females.

### Age specific distribution of burden of CRC in Africa

**Fig. 1:**
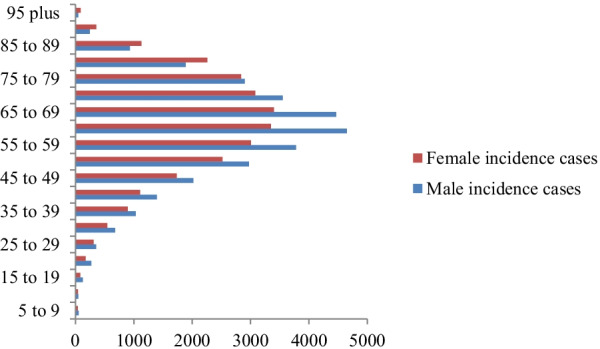
Age specific incidence cases of colorectal cancer among sexes in Africa, 2019

**Fig. 2:**
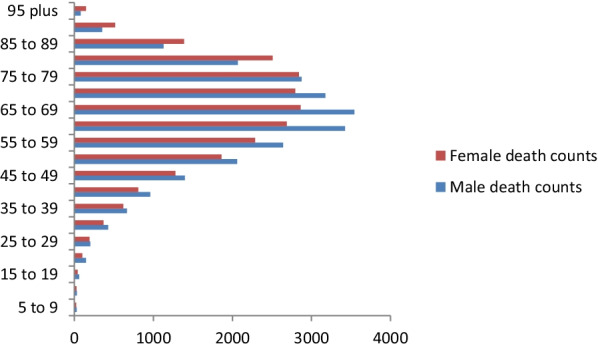
Age specific death counts of colorectal cancer in among sexes in Africa, 2019

**Fig. 3:**
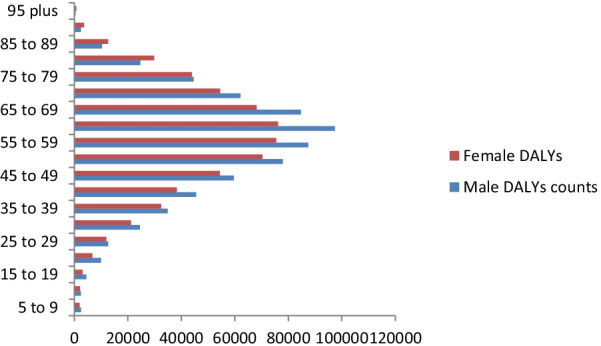
Age specific DALYs counts of colorectal cancer among sexes in Africa, 2019

In 2019, age specific incidence CRC was peaking at 60–69 years in both males and females. Age specific death counts were peaking at 60–69 years while 65–79 years in females. Most DALYs counts were recorded in 55–64 years in both male and female (Figs. 1, 2, 3).

### National burden

There were marked variations of burden of colorectal cancer at national level from 2010 to 2019 in Africa. In 2019, highest estimated new incident cases of colorectal cancer observed in Nigeria 7080 (5310–8960), Egypt 6520 (4680–9010), South Africa 5570 (5000–6290), Algeria 3410 (2670–4280), Morocco 3210 (2390–4070), and Ethiopia 3200 (2400–4460) while lowest new incident cases observed in Sao Tome and Principe 16 (11–22), Seychelles 38 (34–44), Comoros 40 (30–60), and Gambia 50 (50–90). In 2019, highest age-standardised new incidence rate of colorectal recorded in Seychelles 35.7 (31.4–40.6), Mauritius 19.8 (16.1–24.2), Botswana 18.8 (13.5–24.5), and Libya 17 (12.4–21.8) per 100,000 while lowest age-standardised rate saw in Central African Republic 6.3 (4.6–8.7), Malawi 6.3 (4.9–7.8), Niger 5.6 (4.2–7.6), and Somalia 5 (3.1–9.2) per 100,000 (Table 2). From 2010 to 2019, highest percentage change of incidence cases of CRC have seen in Djibouti 77% (40–125), Cabo Verde 77% (39–112%), Rwanda 72% (42–109%), Angola 68% (36–114%), and Democratic Republic of the Congo 63% (29–104%) while lowest changes observed Eswatini 20% (− 7 to 61%), Guinea 19% (− 2 to 19%) and Central African Republic 16% (− 8 to 45%). From 2010 to 2019, highest increased age-standardised incidence rate of CRC has seen in Cabo Verde 48% (15–78), Morocco 25% (1–54%), Sao Tome and Principe 22% (2–42%), Sudan 22% (0–48), Ethiopia 21 (− 1 to 42%), whereas decreased age-standardised incidence rate of CRC has seen Somalia—1% (− 19 to 19), Eswatini—2% (− 22 to 28%), South Africa—5% (− 15 to 8%), Central African Republic—8% (− 25 to 13%), Libya—8% (− 33 to 19%) (Table 3).

**Table 2:** Incidences case, Deaths, and DALYs of colorectal cancer in 2019

Location	Incidence case counts			Age-standardised incidence rate			Death counts		
	2019	95% UI		2019	95% UI		2019	95% UI	
Africa	58,000	52,000	65,000	8.7	8	9.4	49,000	43,000	54,000
Algeria	3410	2670	4280	10.5	8.3	13	2380	1890	2950
Angola	1080	830	1390	10	8.1	12.5	950	740	1210
Benin	360	280	470	7.8	6.3	9.8	330	260	420
Botswana	250	170	330	18.8	13.5	24.5	190	130	250
Burkina Faso	640	500	820	7.4	5.9	9.4	580	460	740
Burundi	330	240	480	7.3	5.3	10.4	300	220	440
CÃ´te d'Ivoire	950	720	1200	9.6	7.6	11.9	850	660	1070
Cabo Verde	60	50	70	13.4	10.7	15.7	50	40	60
Cameroon	1270	930	1680	11.2	8.6	14.6	1110	840	1460
Central African Republic	140	100	190	6.3	4.6	8.7	130	90	180
Chad	390	300	510	7.3	5.7	9.4	370	290	480
Comoros	40	30	60	9	6.6	11.4	40	30	50
Congo	300	220	400	11.9	9.1	15.4	270	200	350
Democratic Republic of the Congo	2190	1480	3260	6.4	4.2	9.6	2000	1340	2960
Djibouti	70	50	100	11.9	9.1	15.8	60	40	80
Egypt	6520	4680	9010	9.8	7.1	13.4	4560	3300	6270
Equatorial Guinea	70	50	110	15.6	9.8	22.4	60	40	90
Eritrea	280	210	370	10.3	8.1	13.2	250	190	320
Eswatini	80	50	110	14.4	9.8	19.7	70	50	100
Ethiopia	3200	2400	4460	7.7	5.8	10.7	2850	2130	4000
Gabon	170	120	210	16.4	12.1	20.3	140	100	180
Gambia	60	50	90	6.8	5	9.2	60	40	80
Ghana	1490	1160	1900	9.5	7.6	11.9	1270	1000	1610
Guinea	390	300	510	7.3	5.6	9.4	370	280	480
Guinea-Bissau	70	50	80	9.3	7.1	11.7	60	40	70
Kenya	1780	1420	2210	8.2	6.7	10	1630	1280	2040
Lesotho	150	100	190	12	8.8	15.5	130	100	170
Liberia	130	90	190	6.8	4.7	9.7	120	80	170
Libya	900	660	1180	17	12.4	21.8	620	450	800
Madagascar	800	580	1080	7.3	5.4	9.7	710	530	960
Malawi	440	340	560	6.3	4.9	7.8	400	310	510
Mali	680	530	850	8.1	6.4	10.1	610	490	770
Mauritania	170	130	220	8.8	6.8	11.1	160	120	200
Mauritius	340	280	420	19.8	16.1	24.2	210	180	260
Morocco	3210	2390	4070	10.3	7.7	13	2480	1860	3110
Mozambique	900	650	1190	8.7	6.4	11.4	830	610	1090
Namibia	130	100	170	9.7	7.7	12.3	110	90	140
Niger	410	290	560	5.6	4.2	7.6	370	280	510
Nigeria	7080	5310	8960	8.9	6.9	11	6380	4880	8140
Rwanda	550	420	700	9.3	7.4	11.7	480	380	610
Sao Tome and Principe	16	11	22	16.5	12	22.2	14	10	19
Senegal	630	500	800	8.9	7.2	11.1	590	470	730
Seychelles	38	34	44	35.7	31.4	40.6	26	23	30
Sierra Leone	240	190	320	7.1	5.5	9.1	220	170	290
Somalia	330	210	620	5	3.1	9.2	310	200	580
South Africa	5570	5000	6290	12.9	11.6	14.5	4600	4160	5200
South Sudan	370	240	550	9.9	6.6	14.7	350	220	530
Sudan	1560	1110	2340	8.2	6	12.3	1260	900	1850
Togo	280	200	370	8.2	6.1	10.5	250	180	320
Tunisia	1800	1300	2440	14.5	10.5	19.5	1150	840	1550
Uganda	1740	1350	2150	12.3	9.8	14.8	1520	1190	1860
United Republic of Tanzania	2460	1930	3180	10.1	8.1	12.7	2180	1730	2790
Zambia	920	670	1200	13.6	10.1	17.4	790	580	1020
Zimbabwe	930	710	1180	13.8	10.6	17.2	820	620	1030

Location	Age-standardised death rate			DALYs counts			Age-standardised DALYs rate		
	2019	95% UI		2019	95% UI		2019	95% UI	
Africa	8.1	7.4	8.8	1,300,000	1,140,000	1,460,000	180	160	200
Algeria	8	6.4	9.8	57,600	45,200	72,400	170	130	210
Angola	9.7	7.8	12	27,800	20,700	35,900	220	170	280
Benin	7.6	6.2	9.5	8500	6500	11,300	160	130	210
Botswana	15.8	11.6	20.5	5200	3500	7100	350	240	460
Burkina Faso	7.3	5.8	9.1	15,400	11,900	19,900	160	120	200
Burundi	7.2	5.2	10.1	8900	6200	12,900	170	120	240
CÃ´te d'Ivoire	9.4	7.6	11.5	23,400	17,400	30,300	200	160	250
Cabo Verde	11.4	9	13.4	1000	800	1100	220	180	260
Cameroon	10.6	8.3	13.8	30,000	21,600	40,900	230	170	300
Central African Republic	6.4	4.7	8.8	4000	2800	5600	160	110	220
Chad	7.4	5.8	9.3	9800	7400	13,000	160	120	210
Comoros	8.6	6.4	10.8	1000	700	1300	190	140	250
Congo	11.4	8.8	14.5	7500	5300	10,200	260	190	340
Democratic Republic of the Congo	6.2	4.1	9.5	56,300	38,100	83,800	140	100	210
Djibouti	11.2	8.7	14.7	1700	1200	2500	250	180	350
Egypt	7.4	5.4	10.1	133,000	95,300	183,300	180	130	250
Equatorial Guinea	14.2	9.1	20	1700	1000	2500	310	190	450
Eritrea	10	7.9	12.7	7600	5700	10,100	240	180	310
Eswatini	13.5	9.3	18.2	1900	1300	2700	300	200	430
Ethiopia	7.3	5.5	10.4	79,000	58,500	109,700	170	120	240
Gabon	14.9	11.3	18.2	3700	2600	4800	330	240	420
Gambia	6.6	4.8	8.8	1500	1000	2000	140	100	190
Ghana	8.8	7	10.9	34,900	26,500	45,100	190	150	250
Guinea	7.2	5.5	9.2	9400	7000	12,400	160	120	210
Guinea-Bissau	9.1	6.9	11.3	1700	1200	2200	210	150	260
Kenya	8.1	6.5	10	45,300	35,300	57,200	180	140	230
Lesotho	11.7	8.7	15.1	3600	2500	4800	270	190	350
Liberia	6.7	4.6	9.5	3200	2100	4500	140	100	200
Libya	12.5	9.1	15.8	17,100	12,300	22,500	300	220	390
Madagascar	7.1	5.3	9.3	21,600	15,500	29,000	170	120	220
Malawi	6.1	4.7	7.5	10,700	7900	13,900	130	100	170
Mali	7.8	6.3	9.7	16,300	12,500	21,000	170	140	220
Mauritania	8.3	6.5	10.3	3600	2600	4700	170	120	210
Mauritius	12.8	10.5	15.5	5100	4100	6200	290	240	360
Morocco	8.5	6.3	10.5	63,600	47,400	81,100	190	150	250
Mozambique	8.7	6.5	11.2	22,400	15,800	29,800	190	140	250
Namibia	8.7	7	10.8	2800	2100	3700	190	150	240
Niger	5.6	4.2	7.5	10,100	7200	14,000	120	90	160
Nigeria	8.6	6.7	10.8	157,300	116,500	205,200	170	130	220
Rwanda	8.8	7.1	10.8	13,300	10,000	17,500	200	150	250
Sao Tome and Principe	15.2	11.2	20.6	400	200	500	320	230	430
Senegal	8.7	7.1	10.8	14,400	11,000	18,600	180	140	230
Seychelles	25.3	22.2	28.7	600	500	700	550	490	630
Sierra Leone	7	5.4	8.9	5800	4300	7600	150	110	190
Somalia	5	3.2	9.3	9600	6100	17,900	120	80	230
South Africa	11.2	10.1	12.6	111,500	100,300	126,600	240	220	270
South Sudan	10	6.6	14.8	9500	5900	14,700	220	140	340
Sudan	7.1	5.3	10.6	34,700	24,000	50,900	160	120	240
Togo	7.9	5.9	10	6700	4800	9000	170	120	220
Tunisia	9.7	7.2	13	26,500	19,100	36,100	210	150	280
Uganda	11.6	9.2	13.8	43,100	32,500	54,500	270	210	330
United Republic of Tanzania	9.5	7.7	11.8	59,000	45,100	78,200	220	170	280
Zambia	12.6	9.5	16	23,300	16,600	30,700	300	220	380
Zimbabwe	12.9	9.9	16.2	22,800	17,100	29,300	300	220	370

**Table 3:** Percentage changes of national incidence cases, deaths and DALYs in Africa from 2010 to 2019

Location	Incidence cases			ASIR			Death counts		
	Value (%)	95% UI (%)		Value (%)	95% UI (%)		Value (%)	95% UI (%)	
Africa	48	34	62	11	1	21	41	28	55
Algeria	57	24	98	10	− 12	38	43	14	79
Angola	68	36	114	14	− 4	43	63	31	105
Benin	42	19	70	3	− 12	21	40	19	65
Botswana	51	19	92	9	− 11	36	41	12	77
Burkina Faso	59	35	92	18	2	39	55	32	85
Burundi	40	15	73	0	− 17	21	39	15	69
CÃ´te d'Ivoire	39	11	73	4	− 14	25	38	12	69
Cabo Verde	77	39	112	48	15	78	65	28	100
Cameroon	47	18	83	6	− 14	29	41	14	75
Central African Republic	16	− 8	45	− 8	− 25	13	16	− 8	45
Chad	40	15	68	6	− 11	27	37	14	63
Comoros	44	17	75	10	− 10	32	40	14	68
Congo	53	23	90	9	− 11	33	49	20	84
Democratic Republic of the Congo	63	29	104	20	− 4	49	58	26	97
Djibouti	77	40	125	13	− 8	39	71	35	115
Egypt	59	18	110	20	− 9	56	45	9	89
Equatorial Guinea	60	20	120	13	− 13	52	53	16	105
Eritrea	47	21	79	9	− 8	32	43	19	73
Eswatini	20	− 7	61	− 2	− 22	28	16	− 9	55
Ethiopia	62	33	92	21	− 1	42	56	30	83
Gabon	37	9	72	7	− 13	32	31	5	63
Gambia	47	15	81	13	− 10	39	43	13	75
Ghana	53	26	86	14	− 5	35	48	22	77
Guinea	19	− 2	46	7	− 11	29	16	− 4	40
Guinea-Bissau	29	6	57	2	− 15	22	27	5	54
Kenya	53	29	79	9	− 7	27	46	26	69
Lesotho	25	− 2	57	12	− 11	40	20	− 5	50
Liberia	34	8	68	3	− 16	27	31	6	63
Libya	35	− 3	76	− 8	− 33	19	35	− 1	75
Madagascar	47	17	84	5	− 15	30	44	16	79
Malawi	38	11	67	3	− 15	22	36	10	62
Mali	42	18	70	7	− 10	28	38	16	65
Mauritania	41	10	71	8	− 14	29	35	8	63
Mauritius	54	23	90	16	− 6	43	47	20	80
Morocco	61	31	99	25	1	54	48	21	82
Mozambique	44	12	82	15	− 8	44	40	11	77
Namibia	47	18	81	18	− 4	44	37	12	68
Niger	57	30	91	9	− 8	28	55	29	86
Nigeria	41	6	84	8	− 17	38	38	7	82
Rwanda	72	42	109	17	− 2	39	67	38	100
Sao Tome and Principe	50	23	74	22	2	42	40	17	63
Senegal	46	19	78	13	− 7	36	42	17	72
Seychelles	49	29	69	15	0	31	37	19	55
Sierra Leone	48	20	82	10	− 9	34	42	17	74
Somalia	35	8	66	− 1	− 19	19	34	9	64
South Africa	22	8	39	− 5	− 15	8	16	4	32
South Sudan	24	1	55	1	− 16	25	23	0	54
Sudan	57	28	93	22	0	48	45	20	80
Togo	53	25	88	7	− 12	27	49	22	81
Tunisia	53	18	101	13	− 12	48	38	8	79
Uganda	54	25	88	12	− 7	34	51	23	81
United Republic of Tanzania	50	22	79	12	− 7	33	46	21	74
Zambia	63	29	101	11	− 11	36	53	22	90
Zimbabwe	29	4	61	4	− 15	29	27	3	58

Location	ASDR			DALYS counts			Age-standardised DALYS rate change		
	Value (%)	95% UI (%)		Value (%)	95% UI (%)		Value (%)	95% UI (%)	
Africa	6	− 3	16	41	27	56	6	− 5	16
Algeria	1	− 19	24	39	9	76	− 1	− 23	26
Angola	11	− 8	36	59	25	101	7	− 17	41
Benin	2	− 12	19	41	16	71	5	− 14	27
Botswana	3	− 16	27	40	8	80	5	− 23	45
Burkina Faso	16	0	35	61	34	98	21	0	50
Burundi	− 1	− 17	18	40	14	74	0	− 20	26
CÃ´te d'Ivoire	3	− 14	22	36	6	71	7	− 14	34
Cabo Verde	41	8	70	62	30	94	19	− 4	47
Cameroon	2	− 16	24	41	12	81	3	− 20	33
Central African Republic	− 8	− 25	13	15	− 9	45	− 8	− 32	23
Chad	5	− 11	25	38	14	68	8	− 12	34
Comoros	7	− 11	28	45	12	78	10	− 16	36
Congo	7	− 13	30	47	15	87	0	− 24	31
Democratic Republic of the Congo	17	− 5	45	60	25	102	16	− 9	49
Djibouti	9	− 11	32	66	28	116	8	− 17	38
Egypt	10	− 15	41	46	9	92	6	− 20	37
Equatorial Guinea	9	− 15	46	53	11	114	− 1	− 30	45
Eritrea	7	− 10	29	41	14	72	9	− 15	38
Eswatini	− 6	− 25	22	11	− 15	51	− 9	− 36	34
Ethiopia	16	− 4	36	54	27	81	13	− 8	38
Gabon	3	− 15	25	31	2	68	− 4	− 27	27
Gambia	11	− 11	34	44	11	80	13	− 14	45
Ghana	10	− 8	29	46	18	81	7	− 14	31
Guinea	5	− 12	25	22	− 1	49	6	− 15	32
Guinea-Bissau	1	− 15	21	26	3	57	2	− 18	28
Kenya	5	− 9	21	44	23	71	7	− 13	35
Lesotho	9	− 13	35	23	− 5	56	9	− 23	47
Liberia	3	− 15	24	36	7	72	4	− 17	30
Libya	− 8	− 32	18	35	− 5	77	− 10	− 38	18
Madagascar	4	− 15	27	45	15	82	6	− 17	35
Malawi	2	− 15	20	36	7	68	4	− 20	30
Mali	5	− 11	24	41	15	73	4	− 16	27
Mauritania	4	− 15	24	31	0	64	0	− 22	23
Mauritius	10	− 10	34	38	11	71	11	− 10	37
Morocco	17	− 4	44	45	17	78	5	− 16	31
Mozambique	13	− 9	41	42	9	82	15	− 15	57
Namibia	11	− 8	35	37	8	72	10	− 18	45
Niger	8	− 9	25	55	26	90	10	− 9	34
Nigeria	7	− 16	37	39	4	91	9	− 25	62
Rwanda	14	− 4	34	63	32	100	13	− 8	39
Sao Tome and Principe	17	− 1	35	48	20	75	9	− 11	30
Senegal	10	− 8	32	43	13	78	13	− 12	42
Seychelles	8	− 5	22	40	22	60	6	− 13	28
Sierra Leone	7	− 10	29	45	17	83	10	− 12	38
Somalia	− 2	− 18	17	33	8	64	0	− 20	27
South Africa	− 9	− 17	3	13	0	30	− 10	− 24	7
South Sudan	0	− 17	23	22	− 3	58	2	− 17	34
Sudan	15	− 5	39	47	17	86	10	− 14	40
Togo	4	− 13	24	46	17	82	8	− 14	33
Tunisia	2	− 20	32	35	4	79	0	− 25	33
Uganda	10	− 9	30	51	19	87	14	− 11	46
United Republic of Tanzania	10	− 7	29	47	17	81	14	− 8	40
Zambia	6	− 15	29	55	19	97	7	− 18	38
Zimbabwe	3	− 16	28	29	3	64	3	− 23	40

In terms of death counts in both sexes, Nigeria, South Africa, Egypt, and Ethiopia were the leading four countries with 6380 (4880–8140), 4600 (4160–52), 4560 (3300–6270), and 2850 (2130–4000) deaths respectively in 2019. Comoros 40 (30–50), Seychelles 26 (23–30), and Sao Tome and Principe 14 (10–19) had lowest death counts in 2019. In 2019, Seychelles 25.3 (22.2–28.7), Botswana 15.8 (11.6–20.5), Sao Tome and Principe 15.2 (11.2–20.6), and Gabon 14.9 (11.3–18.2) per 100,000 had a highest age-standardised death rate whereas Democratic Republic of the Congo 6.2 (4.1–9.5), Malawi 6.1 (4.7–7.5), Niger 5.6 (4.2–7.5), and Somalia 5 (3.2–9.3) per 100,000 had a lowest age-standardised death rate (Table 2). From 2010 to 2019, highest percentage change of death counts due to CRC observed in Djibouti 71% (35–115%), Rwanda 67% (38–100%), Cabo Verde 65% (28–100%), Angola 63% (31–105%), Democratic Republic of the Congo 58% (26–97%), and Ethiopia 56% (30–83%) while lowest change observed in Eswatini 16% (− 9 to 55%), Guinea 16% (− 4 to 40%), Central African Republic 16% (− 8 to 45%), and South Africa 16% (4–32%). Cabo Verde 41% (8–70%), Democratic Republic of the Congo 17% (− 5 to 45%), Morocco 17% (− 4 to 44%) had highest percentage change of age-standardised death rate, while Burundi—1% (− 17 to 18%), Somalia—2% (− 18 to 17%), Eswatini—6% (− 25 to 13%), Central African Republic—8% (− 25 to 13%), Libya—8% (− 32 to 18%), and South Africa—9% (− 17 to 3%) had decreased age-standardised death rate from 2010 to 2019 (Table 3).

In 2019, DALYs counts due to CRC in Africa were ranging from 400 to 157, 3000. The four leading countries in terms of DALYs counts in both sexes were Nigeria 157,300 (116,500–205,200), Egypt 133,000 (95,300–183,300), South Africa 111,500 (100,300–126,600), and Ethiopia 79,000 (58,500–109,700) while Comoros, Seychelles, and Sao Tome and Principe had lowest DALYs counts with 1000 (700–1300), 600 (500–700) and 400 (200–500) respectively in 2019. In 2019, Seychelles 550 (490–630), Botswana 350 (240–460), Gabon 330 (240–420), Sao Tome and Principe 320 (230–430), and Equatorial Guinea 310 (190–450) per 100,000 had highest DALYs counts, whereas Malawi 130 (100–170), Niger 120 (90–160), and Somalia 120 (80–230) per 100,000 had lowest DALYs counts in Africa (Table 2). From 2010 to 2019, Djibouti 66% (28–116%), Rwanda 63% (32–100%), Cabo Verde 62% (30–94%), Burkina Faso 61% (34–98%), and Democratic Republic of the Congo 60% (25–102) had highest percentage change DALYs counts, while Central African Republic 15% (− 9 to 45%), South Africa 13% (0–30%), and Eswatini 11% (− 15 to 51%) lowest percentage of DALYs counts in Africa. From 2010 to 2019, Decreased age-standardised DALYs rate was observed in Algeria—1% (− 23 to 26%), Equatorial Guinea—1% (− 30 to 45%), Gabon—4% (− 27 to 27%), Central African Republic—8% (− 32 to 23%), Eswatini—9% (− 36 to 34%), Libya—10% (− 38 to 18%), and South Africa—10% (− 24 to 7%) (Table 3).

## Discussion

From 2010 to 2019, age-standardised rates and counts of incidence cases, deaths, and DALYs of colorectal cancer in Africa increased with heterogeneous trend across the nations. The absolute numbers of incidence cases of CRC have increased in Asia, America, and Europe as well as worldwide. In addition to this, age standardised incidence rate of CRC also raised from 2010 to 2019 globally and in all regions except in Europe. Changes of incidence cases ranged from 16% in Central African Republic to 77% in Djibouti. More than 90% of countries had increased age-standardised incidence rate, however, decreased age-standardised incidence rate observed in Somalia, Eswatini, South Africa, Central African Republic, and Libya. This trend of CRC has attributed to population growth, aging, changing risk factors, adopting screening, increasing diagnosis, and registration of colorectal cancer mainly in Africa and Asia. Increased absolute incident cases and age-standardised incidence rate of CRC indicates that change in environmental, demographic, epidemiological, and sociodemographic have played a significant role in rising of burden of colorectal cancer in Africa. More than 55% [6] of colorectal cancer can be prevented with evidence based modification of strong modifiable risk factors such as smoking [13], weight gain [14], alcohol consumption [15], and lack of physical inactivity [16] and unhealthy diet. Change of living standards in transition countries in North Africa has exposed new risk factors such as sedentary life and metabolic syndrome. The colorectal cancer has a male predilection with peaking 60–69 years; however, the disparity is not much as western. This might be due to males have higher prevalence rates of modifiable risk factors such as smoking [17], alcohol consumption [18] and protective effect of estrogen for CRC in females [19].

From 2010 to 2019, we found that death counts and age-standardised death rates of CRC have increased in Africa. Increased death counts were also observed in America, Asia, Europe and globally. However, trend of age-standardised death rate of CRC has decreased in America, Europe and global as whole with slight stable change in Asia. From 2010 to 2019, heterogeneity trend and burden of CRC mortality has noticed across nations of Africa. Mortality CRC related has increased significantly, ranging from 16 to 71% with more than 90% of countries had increased age-standardised death rate, however, decreased age-standardised incidence rate was observed only in Burundi, Somalia, Eswatini, South Africa, Central African Republic, and Libya. Increased absolute colorectal cancer related mortality and age-standardised death rate have associated to increased population size and change age structure, decreasing mortality from other disease, increased risk factors, low rate of screening, diagnosis, and, treatment in Africa. There are a strong evidence described that mortality and incidence of colorectal cancer can be reduced through screening. Apply primary, secondary and tertiary prevention modality such as reduction of modifiable risk factors and adopting evidence based screening modality are key steps to achieve sustainable development goals [10] and 25 by 25 targets [9] of colorectal cancer.

DALYs measurement is an important indicator of quality of CRC cares. Results from this study revealed that absolute DALYs counts and age-standardised rates of CRC have increased between 2010 and 2019 in Africa. Increased DALYs counts of colorectal cancer is a global phenomenon, however, change in Africa as compared with Asia, America, Europe, and global as a whole was invariably significant. Despite regions and global have increased DALYs counts of CRC between 2010 and 2019, trend of age-standardised DALYs rate of CRC was decreasing in America, Europe and global as whole with slight stable in Asia. Most of DALYs was contributed from YLL in Africa, which indicates low survival rate. Increased age-standardised rate of death and DALYs of colorectal cancer indicates low efforts and progresses for CRC standard and qualitive care-early diagnosis and treatment, primary prevention of modifiable risk factors and implementation of secondary prevention modality in Africa and across most nations. This serious effect would be due to poor cancer infrastructure and policy, low workforce capacity, cancer center to diagnosis and treatment, low finical security and low of universal health coverage in Africa. Geographical variation of screening of CRC has attributed to geographic variation in CRC incidence, ability in identify the target population at risk, economic resource, human resource capacity, health care structure, infrastructure, and health care policy and direction [20]. Evidence from mathematical modeling study recommended that colonoscopy screen in Africa begins at age of 50 years [21]. Estimated efficacy of colorectal screening ranged from 2.6% (single screen with fecal occult blood test) to over 50% (such as colonoscopy every 10 years, or annual fecal occult blood test and sigmoidoscopy every 5 years) [21]. However, recommendation of population based CRC screen in Africa is questionable due high burden of communicable disease, low human capacity, availability of colonoscopy, and relatively low burden of CRC as compared as other health condition [22]. Several factors might have contributed to low rate of quality of CRC care in Africa such as inaccessibility of screening [20], early detection, low quality and skill in oncological surgery, inaccessibility of radiotherapy, chemotherapy, target therapy and palliative therapy [23].

### Limitation

GBD studies provide qualitive, compressive, and up-dated evidence of global, regional and national burden of diseases for policy maker, researcher and planner. This study has played a great and invaluable role, particularly for Africa. The main limitation of this study is unavailability and quality of data sources. Therefore, African nation should have improved cancer registration, collaborated and provided data to IHME, and follow the prediction and give feedback.

## Conclusion

Increased age-standardised rate of incidence, death and DALYs have been observed in Africa and across a nations. Evidence from this analysis showed that there is fast rising burden of colorectal cancer due to increased prevalence of modifiable risk factors such as smoking, alcohol, unhealthy diet, sedentary lifestyle, and metabolic syndrome. Observation indicates that there are low efforts and progresses in CRC standard and qualitative care-evidence based early diagnosis and treatment, primary prevention of modifiable risk factors and implementation of secondary prevention modality. This alarm all nations and global community to call integrated, comparative and resilience measures for prevention, awareness creation, adopting screening, and evidence based treatments and rehabilitations.

## Data Availability

Data are available in GBD 2019 tools (http://ghdx.healthdata.org/gbd-results-tool).

## Abbreviations

AAPC: Annual average percentage change
ASDR: Age-standardized death rate
ASIR: Age-standardized incidence rate
CRC: Colorectal cancer
GBD: Global Burden of Disease
IHME: Institute of Heath Metrics and Evaluation
WHO: World Health Organization
